# Effects of germination and ultrasound treatment on the thermodynamics, nutritional and structural quality of highland barley fractions

**DOI:** 10.1016/j.ultsonch.2025.107652

**Published:** 2025-10-27

**Authors:** Tabussam Tufail, Huma Bader Ul Ain, Jawad Ashraf, Farhan Saeed, Zunaira Basharat, Zahoor Ahmed, Muhammad Waseem, Bin Xu, Muhammad Faisal Manzoor, Robert Mugabi

**Affiliations:** a College of Pharmaceutical Sciences, Zhejiang University of Technology, Hangzhou, China; b School of Food and Biological Engineering, Jiangsu University, Zhenjiang, Jiangsu 212013, China; c University Institute of Diet and Nutritional Sciences, The University of Lahore, Pakistan; d School of Food Science and Engineering, Yangzhou University, Yangzhou, China; e College of Food Science and Technology, Bohai University, Jinzhou, Liaoning, China; f Department of Food Sciences, Government College University Faisalabad, Pakistan; g College of Food and Nutrition, Anhui Agricultural University, Hefei 230036, China; h Human Nutrition and Dietetics, School of Food and Agricultural Sciences, University of Management and Technology, Lahore, Pakistan; i Department of Food Science and Technology, Faculty of Agriculture and Environment, Islamia University of Bahawalpur, Pakistan; j Guangdong Provincial Key Laboratory of Intelligent Food Manufacturing, School of Food Science and Engineering, Foshan University, Foshan, China; k Faculty of Sciences and Technology, ILMA University, Karachi, Pakistan; l Department of Food Technology and Nutrition, Makerere University, Kampala, Uganda

**Keywords:** Highland barley, Ultrasound, Germination, GABA, Phenolics, Gene expression

## Abstract

Current research investigates the effect of ultrasonication (US) (20/40/60 kHz, 220 W, 30 min), germination (65 °C, 6 h), and their combined treatment (US + G) on gamma-aminobutyric acid (GABA) enhancement and quality profile of barley flour and bran. The results showed the highest improvements in ultrasound-assisted germinated barley flour. GABA levels increased significantly, correlating with enhanced GAD and GABA-T enzyme activities. Similarly, TPC, TFC and antioxidant potential were improved, associated with upregulated expression of mPAL, mC3H, mCHS, and mC4H genes in WB and BB tissues, enhancing phenolics biosynthesis. Surface disruptions, increased porosity, and cellular disintegration were observed in ultrasonicated samples. XRD patterns showed significant molecular arrangements and increased amorphous regions in ultrasound-treated fractions. Furthermore, FTIR spectra reveal protein unfoldings in the amide I region, suggesting enhanced protein functionality in ultrasound-assisted germinated flour. Hence, ultrasound-assisted germination can be proposed as a sustainable approach for nutritional enhancement of barley fractions to improve their suitability for functional implications.

## 1 Introduction

Barley (*Hordeum vulgare),* one of the oldest cereal crops, ranks as the fourth most produced grain globally, accounting for approx. 12 % of total cereal production after wheat, rice, and corn. Naked caryopsis barley thrives predominantly on Tibetan Plateau elevations ranging from 1400 to 4700 m [1]. Barley is notably rich in functional compounds, including β-glucan, arabinoxylan, and polyphenols linked to its antioxidant, anticancer, and antibacterial properties [2]. Furthermore, it contains significant levels of GABA, a nonprotein amino acid recognized as an essential human inhibitory neurotransmitter responsible for anti-hypertensive, hypoglycemic, and tranquillizing effects [3].

GABA biosynthesis and metabolism are regulated through *GABA shunt* and *polyamine degradation pathways*, the former being plants' main GABA accumulation route. The GABA shunt pathway involves GABA production through the decarboxylation of glutamate-mediated by GAD. Subsequently, GABA is metabolized to succinic semialdehyde by GABA transaminase [4]. GABA, the inhibitory neurotransmitter of the central nervous system, enhances neuronal development, improves insomnia, depression, and anxiety, and confers a calming effect [5]. Generally, GABA levels in grains are lower compared to other foods. However, various processing treatments, including microbial fermentation, salt elicitation, heat treatment, germination, ultrasonication, exposure to UV light, high-pressure, and pulsed electric field, improve GABA levels by facilitating its release and enhancing enzyme activity.

Germination and fermentation are traditional approaches used for enzyme activation, improving grain digestibility, and phytochemical [6] profile. Furthermore, grain germination elevates the availability of free amino acids, stimulates GABA accumulation, and causes nutritional enhancement by increasing minerals, dietary fiber, and phenolic compounds [7]. Ultrasound (US), an emerging non-thermal processing technology, augments the production of bioactive compounds, including primary and secondary metabolites, in cereal products [8]. The US activates endogenous enzymes, catalyzing antinutrient degradation in cereals and enhancing protein bioavailability and [9] digestibility [10].

Previously, the effect of individual treatments has been studied on highland barley fractions. However, current research explores ultrasound-assisted germination’s synergistic effect on nutritional enhancement, techno-functional properties, and microstructural characteristics of highland barley fractions. The study evaluated GABA accumulation, phenolics profiling, antioxidant activity potential, enzymatic activity, and relative gene expressions of treated barley fractions, aiming to establish a scientific basis for developing functional barley food products demonstrating applications of ultrasound-assisted germination in sustainable cereal processing.

## 2 Materials and methods

The study was conducted at the Food and Biological Engineering School, Jiangsu University, China. Highland barley (*Hordeum vulgare*) was procured from Yangnongpi 9: Jiangsu Ruimu Biotechnology Co., Zhenjiang, China, and stored in zip-lock bags to prevent grain deterioration.

### 2.1 Sample preparation

After cleaning, barley grains were disinfected using H_2_O_2_/NaClO (1 %) solution for 30 mins, then soaking in three volumes of distilled water at 25 °C for 2 h [11]. The disinfected grain samples were coded as illustrated in Table 1 and subjected to the following processing conditions: ultrasound treatment for 30 mins at 20/40/60KHz, 220 W, and 5 *sec*-pulse intervals, germination for 6 h at 65 °C, combined treatment, ultrasound-assisted germination, and soaking for 12 h at 25 °C. The native barley grains (C) were untreated, merely washed and dried seeds. After processing, samples were stored at −80 °C for 24 h and then lyophilized for 48 h. Subsequently, samples were mechanically ground into a fine powder, sifted (120-mesh), and stored in pre-labeled air lock PE bags at −24 °C until further characterization.

**Table 1:** Treatment plan for sample preparation.

**Barley Fraction**	**Sample Code**	**Treatment**
Barley Flour	C1	Untreated Barley (C)
	T1	Tri-frequency ultrasound treatment (US)Frequency: (20/40/60 KHz)
	T2	Germination (GE)
	T3	US + GE
	T4	Soaking (S)
Barley Bran	CB1	Untreated Barley (C)
	TB1	Tri-frequency ultrasound treatment (US)Frequency: (20/40/60 KHz)
	TB2	Germination (GE)
	TB3	US + GE
	TB4	Soaking (S)

### 2.2 Quantification of GABA and GABA activity profiling

#### 2.2.1 Determination of GABA content

The GABA content of treated barley fractions was quantified using the HPLC protocol explained by Tuafail et al. [11]. Briefly, 1 g of each treated sample was extracted with 8 % trichloroacetic acid (25 mL) at 25 °C for 60 mins. The resultant extracts were centrifuged at 5590 × g for 10 mins, and the supernatant was filtered through 0.22 μm syringe filters and injected into an HPLC system (C-18 Zorbax column, UV detection at 254 nm) the mobile phase A is 50 mM/L Sodium acetate buffer; mobile phase B is Acetonitrile. The allowed time of separation of GABA was within 20 min at a constant temperature of 30 °C with a 1.0 mL/min flow rate. GABA content was enumerated against the standard curve and presented as mg GABA/g sample.

#### 2.2.2 Determination of glutamic acid decarboxylase (GAD) activity

GAD activity was determined by quantifying GABA production through enzymatic conversion of glutamate. Briefly, 0.5 g of each treated sample was homogenized with 3 ml ice-cold 70 mM PBS buffer (pH 5.8) supplemented with PLP (0.2 mM), β-mercaptoethanol (2 mM), EDTA (2 mM), and glycerol (10 %). The homogenate was centrifuged (12,000 × g, 30 mins, 4 °C), and the supernatant was incubated with the enzyme at 40 °C. GAD activity U/g fresh weight was expressed as the amount of enzyme producing 1 μmol GABA/h.

#### 2.2.3 GABA-T activity assay

Crude enzyme extract was homogenized with phosphate buffer comprising NAD β-mercaptoethanol. Subsequently, the prepared substrate (1:1 α-ketoglutarate/ GABA (100 µL) incubated at 30°C for 30 mins, was added to the solution, and absorbance was measured at 340 nm using UV–VIS spectrophotometer [12].

### 2.3 Determination of total phenolic contents (TPC), total flavonoid contents (TFC), and their relative mRNA gene expressions

Treated barley samples were extracted with 80 % methanol and centrifuged (10,000 g, 15 mins, 4 °C); the resultant supernatant was used to determine TPC and TFC. TPC was determined using the Folin-Ciocalteu assay, absorbance was measured at 760 nm using a UV–Vis spectrometer, and results were expressed as (mg GAE/g DW) [13].

The TFC of the barley fraction was determined by a modified method described by [14]. Briefly, 1 mL of extract was homogenized with 0.4 mL of H_2_O and 40 μL of 5 % NaNO_2_ and incubated for 5 mins. After that, 60 μl of 10 % AlCl3, 200 μL of NaOH, and 200 μL of H_2_O were added, and absorbance was measured at 510 nm using a SpectraMax i3 spectrophotometer, and TFC was expressed as mg QE/g. Similarly, other relative expressions of genes, including C3H, C4H, mPAL, and CHS, were measured using the methods described above.

### 2.4 Determination of antioxidant activity

#### 2.4.1 Determination of DPPH activity

DPPH activity of treated samples was determined using Mehmood et al. (2025) with a few modifications. Briefly, 95 µL of extracts were allowed to react with 0.4 mL of 6 **×** 10^-5^ mol/L freshly prepared DPPH solution, and absorbance was measured at 517 nm at 0 and 30 min against a 95 % methanol blank. DPPH scavenging activity was calculated using the formula;

$$DPPH\;scavenging\;activity\;\left(\%\right)=\frac{\;Blank\;absorbance-\;Sample\;absorbance}{Blank\;absorbance}\times 100$$

#### 2.4.2 Determination of FRAP activity

Briefly, 25 µL of the extract was mixed with 350 µL of ferric-TPTZ reagent and allowed to react for 10 min. Subsequently, absorbance was measured at 593 nm using a 96-well microplate reader. The FRAP activity was expressed as µmol Fe 2 + equiv/g ^1^ [15].

#### 2.4.3 Determination of ABTS activity

ABTS standard solution was prepared by mixing ABTS (7 mM) with K_2_S_2_O_8_ and allowed to react in the dark overnight. The stock solution was diluted using PBS (pH 7.4) until an absorbance of 0.70 ± 0.02 was achieved. 80 μL of barley extract was mixed with 3.92 mL ABTS working solution in the dark for 5 mins, and absorbance was measured at 734 nm and expressed as μg of Trolox equivalent/ gram of dry weight [16].

#### 2.4.4 Determination of electrolyte leakage and oxygen generation

The oxygen generation and electrolyte leakage of treated barley samples were measured by using the methods explained by [17,18].

### 2.5 Determination of enzyme activity of barley fractions

#### 2.5.1 Determination of POD, PPO, CAT, MDA, and H_2_O_2_ activities

The antioxidant potential of treated barley fractions was further assessed by determining antioxidant enzyme activity. Each sample was homogenized with phosphate buffer for enzyme extraction. Polyphenol oxidase (PPO) activity was measured through quinone formation rate after catechol addition, whereas peroxidase (POD) activity was determined by guaiacol oxidation by taking absorbance at 420 and 470 nm, respectively, using a microplate reader [11].

MDA activity was determined using the method of Ilyasov et al. (2020) with a few modifications. Briefly, pulverized tissue samples were extracted with trichloroacetic acid buffer under acidic conditions, and the pink-colored complex was formed, and absorbance was measured at 532 nm using a spectrophotometer.

Catalase (CAT) activity was measured using the method of [19] with a few modifications. 1 g sample ground in sodium phosphate buffer (0.1 mol/L, pH 7.5, 10 mL) containing 40 g/L polyvinylpyrrolidone and 5 mmol/L dithiothreitol in an ice bath, centrifuged at 6000 × g for 20 mins at 4 °C, 100 μl supernatant was mixed with 2.9 mL H_2_O_2_ solution (20 mmol/l) and incubated at 25 °C for 5 mins. CAT activity (U/g DW) was calculated as a decrease in absorbance by 0.01 at 240 nm/g/min.

#### 2.5.2 Determination of lipase and ACE inhibitory activity

Lipase activity was determined using the p-nitrophenyl butyrate (pNPB) assay. Briefly, the enzyme was incubated with pNPB and the absorbance was measured at 410 nm. The rate of increase in absorbance is directly proportional to the lipase activity. Raymond’s ACE inhibition activity method involves enzyme incubation with hippuric-histidyl-histidine (Hip-His-His). And absorbance was recorded at 228 nm. The inhibitory effect of the test sample was evaluated by comparing the substrate hydrolysis rate in the presence and absence of the sample, with inhibition calculated as a percentage of the control [20].

### 2.6 Morphological characterization of barley fractions (SEM)

The morphological characterization of barley grain cell walls was performed using Merlin scanning electron microscope (SEM). Samples were placed on a taped disc, a gold layer was sprayed on the samples, and a scan was performed at 100x and 200x magnification [21].

### 2.7 X-ray diffraction analysis of barley fraction

The crystallographic structure of treated barley flour and bran was evaluated through X-ray diffraction analysis. The samples were placed on scanning discs and subjected to an X-ray diffractometer (Ultima lV, Rigaku, Japan); X-ray tube current was maintained at 40 kV and 40 mA with a scanning range of 10-60° at a scan speed of 5°/m [22].

### 2.8 Spectral analysis of barley fractions (FTIR)

The treated barley flour and bran samples were analyzed using an ATR-FTIR spectrometer with a germanium crystal (Thermo Nicolet Co., Nicolet iS50, USA). FTIR spectra were recorded over 32 scans at a spectral resolution of 4 cm^−1^, utilizing ATR accessories ranging in wavelength from 4000 to 500 cm^−1^. Spectral data were processed and analyzed through OMNIC 9.0 software (Thermo Fisher Scientific, Waltham, MA, USA). The protein’s secondary structure was also determined via Peak Fit v4.12 software [11].

### 2.9 Texture profile analysis of barley fractions

Texture profiles, including hardness, cohesiveness, springiness, and adhesiveness of barley fractions, were observed using a texture analyzer using a 03-bend ridge. Force was applied to dough-based samples prepared using 10 g of each sample kneaded with 8 mL water. The force split the sample was recorded, and the average was determined to determine texture attributes [23].

### 2.10 Determination of rheological properties of barley fractions

The rheological profile of samples was evaluated using the HAAKE RS6000 rheometer (Thermo Fisher Haake, Karlsruhe, Germany) as described by [24]. A sample suspension 10 % (*w*/*w*) was prepared and placed on a magnetic stirrer for 60 mL, then 1 mL of suspension was placed on a rheometer disk, and silicone oil was applied around the plate to avoid sample loss. The steady-state flow test was performed at a shear rate of 0.1–100 s^−1^, and the dynamic viscoelastic test was conducted at an angular frequency of 0.1–100 rad/s at 1 % strain within the linear viscoelastic region.

### 2.11 Determination of aromatic profile via E-nose

The E-nose system with a multi-sensor array was utilized to analyze volatile organic compounds (VOCs) of treated barley samples, whereas control samples served as baselines. The E-nose system used metal oxide sensors (MOS), sensitive to a broad range of VOCs, including alcohols, aldehydes, ketones, and sulfur compounds, allowing for a comprehensive profile of the samples' aroma. Each sample was analyzed thrice to ensure consistency and sensor data was preprocessed to eliminate noise and baseline drift. Principal Component Analysis (PCA) was applied to reduce the dimensionality of the data, and cluster analysis was used to classify the samples based on their sensor responses. This approach is consistent with previous studies that have successfully employed E-nose technology for food quality and aroma profiling [25,26].

### 2.12 Statistical analysis

The obtained data were statistically analyzed using the Statistical Package Origin-Pro 8.5. The experiment followed a completely randomized design (CRD), and the standard deviation was calculated. Analysis of variance (ANOVA) was conducted to assess the significance levels, followed by the Least Significant Difference (LSD) for post-hoc comparisons.

## 3 Results and discussion

### 3.1 Impact of different treatments on GABA content, GAD, and GABA-T activity of treated barley fractions

**Fig. 1:**
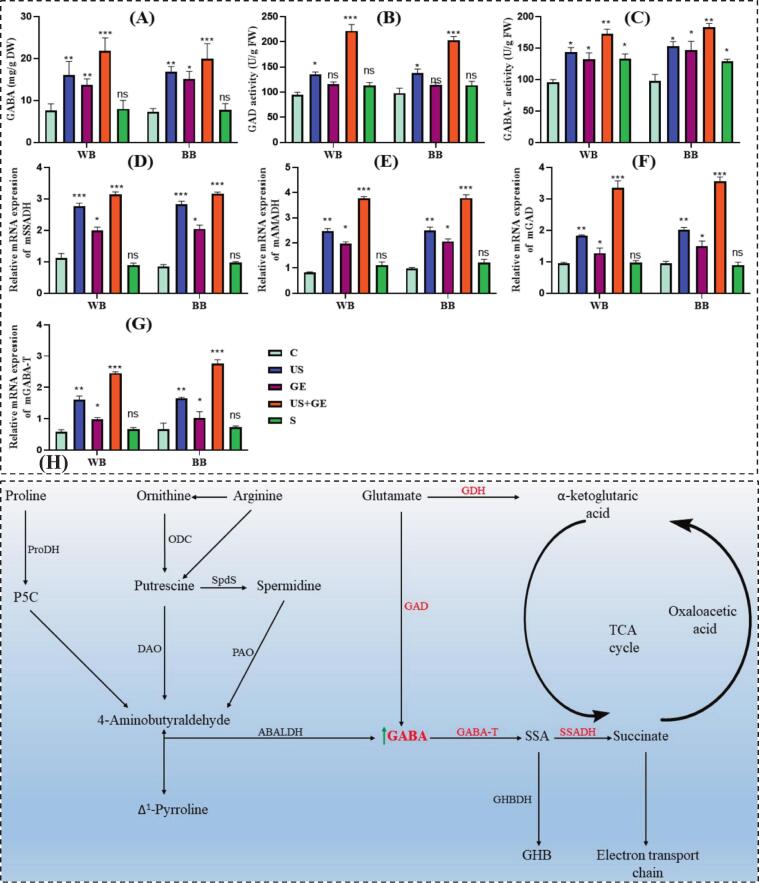
GABA accumulation and its relative gene expression in highland’s barley fractions subjected to ultrasonication, germination, ultrasound assisted germination and soaking. A. GABA contents of treated barley fractions. B. GAD activity of treated barley fractions. C. GABA-T activity of treated barley fractions. D. mRNA expression of mSSADH of treated barley fractions. E. mRNA expression of mAMADH of treated barley fractions. F. mRNA expression of mGAD of treated barley fractions. G. mRNA expression of mGABA-T of treated barley fractions. H. Metabolic pathway of GABA synthesis. C: untreated Tartary buckwheat, US: multifrequency ultrasonication, GE: germination, S: soaking. WB. Whole barley flour. BB. Barley bran. (*p* < 0.01; LSD), (n = 3).

GABA, a four-carbon free amino acid with significant health benefits, showed a prominent increase in both fractions, whole barley flour (WB) and barley bran (BB) treated samples. Germination and ultrasonication enhanced amino acid release, promoting GABA synthesis. Our results suggested a significant increase in GABA in an ordered US + G treatment, followed by US and G, whereas a non-significant (*p* > 0.05) effect of S was observed on GABA. Previously, [27] reported simulated GABA synthesis (18.90  mg/100  g) in ultrasound-assisted germinated wheat, and a similar trend is depicted in the current findings, as shown in Fig. 1A. Similarly, ultrasonication enhanced GABA content in germinating oats [28].

The effect of different treatments on GAD and GABA-T activities of WB and BB is presented in Fig. 1B&C. The figure shows that GAD and GABA-T activities were significantly augmented to 1.5-fold in US and US + G treated samples. In contrast, G and S alone showed a non-significant (*p* > 0.05) impact on barley fractions. GABA accumulation in cereals is regulated by enzymatic activity and gene expression of GABA synthase. In our study, GAD and GABA-T, key enzymes in GABA metabolism, increased significantly on subjection to US and US + GE treatments compared to controls.

### 3.2 Impact of different treatments on activities of GABA synthesis-related enzymes and their gene expression in barley fractions

GAD and GABA-T are key enzymes regulating GABA biosynthesis and metabolism. In weakly acidic cytoplasm, GAD irreversibly converts glutamate to GABA, which is transported to slightly basic mitochondria, where GABA-T induces reversible transformation into SSA [29]. GAD activity is regulated by pH and Ca^2+^/calmodulin (CaM) under slightly acidic (pH < 6.0) conditions; H^+^ enhances GAD activity; however, in normal physiological conditions, Ca^2+^/CaM is the primary activator [30]. Similarly, physical treatment, such as in the US, affects enzyme activity through modulated enzyme conformation [31]. Optimized S treatment regulates enzymatic activity associated with functional compounds' simulated release and synthesis. Previous studies have shown ultrasound-mediated GAD and GABA-T activation in cereals [11,26,32].

The gene expressions of the GABA biosynthesis pathway affected by US, GE, US + GE, and S in both fractions are presented in Fig. 1 (D-G). As shown in Fig. 1 (D-G) in RT-qPCR, gene expression of *mSSADH, mAMADH, mGAD*, and *mGABA-T* was increased (*p* < 0.01) in US, GE, and US + GE treated samples compared to control samples in both fractions. However, S showed a non-significant (*p* > 0.01) effect on the gene expression of both fractions, as presented in Fig. 1 (D-G), indicating the potential of US + GE for possible GABA enhancement in barley fractions. Sonication and hydropriming treatments significantly improve wheat germination while elevating GABA, glutamate, and alanine levels. These treatments also upregulated GAD mRNA expression, supporting improved metabolic balance and cold stress tolerance during germination.

### 3.3 Impact of different treatments on antioxidant activities of barley fractions

The antioxidant potential of treated barley fractions was evaluated through DPPH, ABTS, and FRAP assays; results showed a highly significant increase in all treated samples. Compared to the control, the highest growth was seen in the US + GE treatment as illustrated in Fig. 3 (A-C). Polyphenols are key antioxidants associated with improved antioxidant activities [33], consistent with previous trends that US-assisted germination augments TPC antioxidant capacity in plants and cereal grains [11,34]. [35] determined the enhancement of polyphenols and antioxidant activity in germinated highland barley by ultrasonication, and the results were in line with our current research findings. However, the study investigated the effect of ultrasonic stress germination on polyphenol content, composition, and antioxidant activity in highland barley. Optimal ultrasonic stress germination conditions significantly increased TPC and TFC, enriched key phenolic compounds, and improved antioxidant potency.

### 3.4 Impact of different treatments on the enzyme activity of barley fractions

Obesity is a multifactorial chronic energy imbalance disorder that causes metabolic complications like dyslipidemia and fatty liver disease [36]. Pancreatic lipase (PL) mediates fat breakdown and reduces fat storage, offering a potential obesity management strategy [37].

Our study showed that GABA and polyphenols enhancement in treated barley flour and bran inhibit PL activity, particularly in US + GE treatment, as shown in Fig. 3 (E). Plant extracts have reported similar results linked to their phytochemicals as an effective obesity management therapy [38,39]. ACE catalyzes the conversion of angiotensin I into angiotensin II, a vasoconstrictor that induces vasoconstriction and high blood pressure after binding to its receptor. Various plant extracts have the potential to inhibit ACE activity, thus lowering blood pressure [40]. Many studies showed that GABA-enriched foods, phenolic compounds, and phenolic-rich foods can inhibit ACE activity and, therefore, reduce blood pressure [41]. Fig. 3(D) shows that US + GE showed prominent ACE inhibition in both barley fractions. Highland barley, known for its high content of phenolic compounds such as chlorogenic acid, gallic acid, and caffeic acid, has been linked to significant angiotensin-converting enzyme (ACE) inhibitory activity.

### 3.5 Impact of different treatments on TPC & TFC of barley fractions

**Fig. 2:**
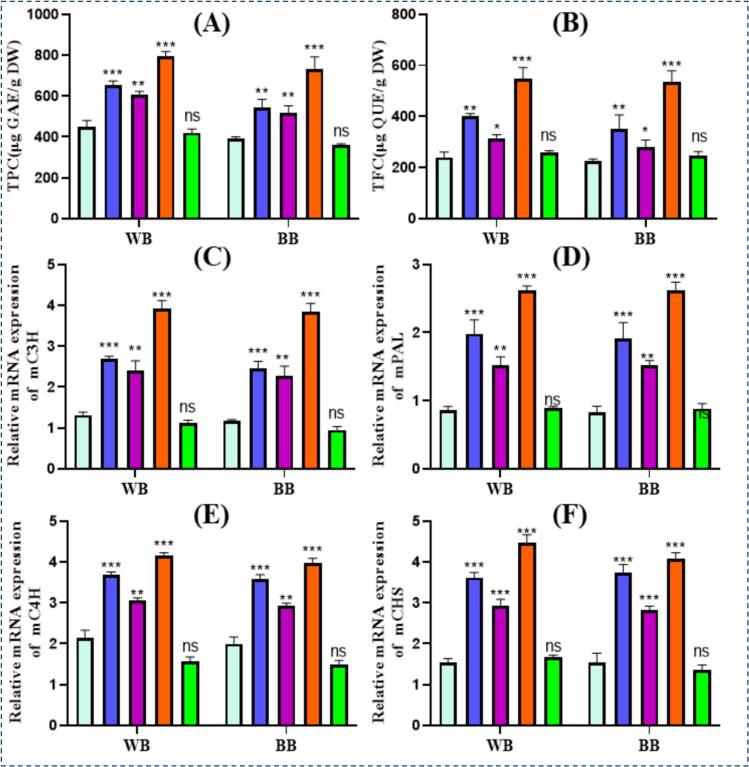
TPC, TFC and their relative gene expression of highland’s barley fractions subjected to ultrasonication, germination, ultrasound assisted germination and soaking. A. TPC of treated barley fractions. B. TFC of treated barley fractions. C. Relative mRNA expression of mC3H gene of treated barley fractions. D. Relative mRNA expression of mPAL gene of treated barley fractions. E. Relative mRNA expression of mC4H gene of treated barley fractions. F. Relative mRNA expression of mCHS gene of treated barley fractions. C: untreated tartary buckwheat, US: multifrequency ultrasonication, GE: germination, S: soaking. WB. Whole barley flour. BB. Barley bran. (*p* < 0.01; LSD), (n = 3).

Phenolics, crucial for developing stress tolerance mechanisms in plants, are also linked with antioxidant and anti-proliferative activities in animals. The effect of different processing treatments on the TPC and TFC of both barley fractions is presented in Fig. 2 (A) and (B). As shown in Fig. 2 (A) and (B), TPC and TFC were increased for all treatments; however, the highest increase was observed in US + GE for both barley fractions, but S showed non-significant results.

**Fig. 3:**
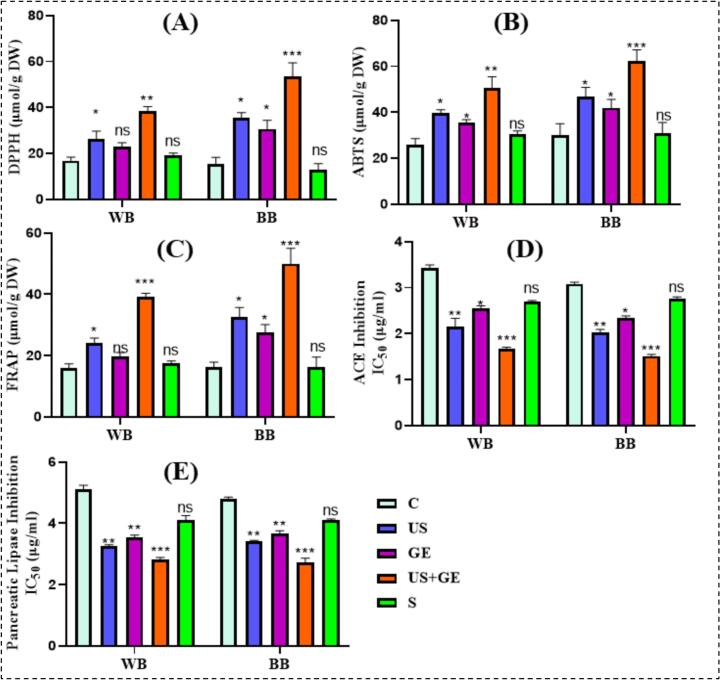
Antioxidant and enzyme activities of highland’s barley fractions subjected to ultrasonication, germination, ultrasound assisted germination and soaking. A. DPPH free radical scavenging activity of treated barley fractions. B. ABTS free radical scavenging activity of treated barley fractions. C. FRAP free radical scavenging activity of treated barley fractions. D. ACE inhibitory activity of treated barley fractions. E. Pancreatic lipase inhibitory activity of treated barley fractions. C: untreated Tartary buckwheat, US: multifrequency ultrasonication, GE: germination, S: soaking. WB. Whole barley flour. BB. Barley bran. (*p* < 0.01; LSD), (n = 3).

The US accelerates hydration by creating pores in the barley seed coat, causing molecular modifications and enzyme catalysis, activating plant defense response, and enhancing secondary metabolites production [42,43]. Furthermore, ultrasonic cavitation and its associated mechanical effect increase cell membrane permeability, facilitating diffusion and transmembrane transportation of ions and metabolites [44]. Furthermore, exogenous GABA permeates the cell membrane, triggering hormonal production and regulating intracellular modifications, upregulating polyphenol metabolism through gene expression. The US-assisted germination enhances flavonoid synthase activity and related gene expression, promoting flavonoid accumulation [33,45]. Therefore, the increased polyphenol content of treated WB and BB can be attributed to US + GE-induced biochemical modifications Fig. 3.

### 3.6 Impact of different treatments on gene expression of PAL, C3H, CHS, and C4H in barley fractions

The phenolic biosynthesis mainly depends on the phenylpropanoid metabolism pathway, involving PAL, C4H, and 4CL as key enzymes [46]; hence, their activities affect the phenolic biosynthesis rate. Relative quantitative analysis of target genes (*mPAL, mC3H, mCHS,* and *mC4H*) was performed using the control group as a reference. Changes in mRNA expression levels of targeted genes in treated WB and BB tissues are presented in Fig. 2 (C-F). A significant (*p* < 0.05) increase in t*mPAL, mC3H, mCHS,* and *mC4H* gene expressions was observed in US, GE, and US + GE treated groups, indicating phenolics biosynthetic pathway activation. The highest expression was shown by US + GE-treated barley fractions, supported by higher antioxidant activity and elevated TPC and TFC in these groups.

Our findings indicate that US, GE, or US + GE treatments activated key enzymes involved in phenylpropanoid metabolism, catalyzing metabolic substrates required to synthesize various phenolic compounds. [47] reported increased phenolics in cherry tomatoes that were ultrasonically washed. The increase was related to PAL and 4CL activation. Furthermore, PCA suggested that stress factors regulate key genes involved in plant stress responses, facilitating metabolic reactions to advance in a regulated manner and increasing TPC [33,48]. Hence, increased TPC and PAL, C4H, C3H, and 4CL activities in WB and BB were attributed to regulated gene expression after ultrasonic and germination.

### 3.7 Impact of different treatments on oxidative stress indicators of barley fractions

**Fig. 4:**
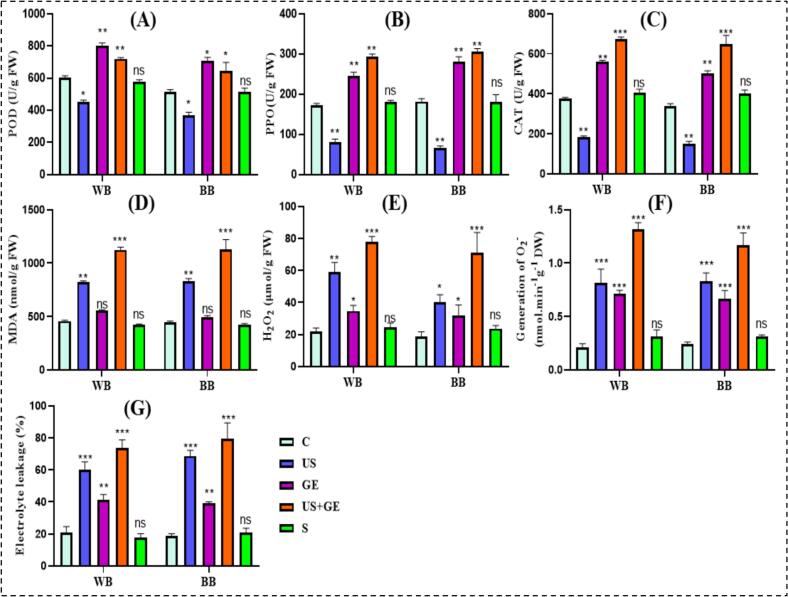
Antioxidant stress indicators of highland’s barley fractions subjected to ultrasonication, germination, ultrasound assisted germination and soaking. A. POD activity of treated barley fractions. B. PPO activity of treated barley fractions. C. CAT activity of treated barley fractions. D. MDA activity of treated barley fractions. E·H_2_O_2_ activity of treated barley fractions. F. O_2_^–^ ions generation of treated barley fractions G. Electrolyte leakage of treated barley fractions C: untreated Tartary buckwheat, US: multifrequency ultrasonication, GE: germination, S: soaking. WB. Whole barley flour. BB. Barley bran. (*p* < 0.01; LSD), (n = 3).

PPO and POD catalyze the enzymatic browning and reduction of the polyphenols. Oxidative stress triggers both enzymatic and non-enzymatic antioxidant defense systems in plants [49]. During low-temperature stress, excessive ROS accumulation stimulates POD activity, scavenging ROS. Similarly, US and GE elicit PPO and POD activity. Different treatments' effects on PPO and barley fractions' POD activity are presented in Fig. 4 (A) and (B). The result showed the highest increase in combined treatment, whereas S showed non-significant variation.

SOD, CAT, and MDA are ROS-scavenging, protecting plant cells from excessive ROS stress. CAT and MDA directly catalyze H_2_O_2_ to water and oxygen. As shown in the Fig. 4 (C) and (D), the trends of CAT and MDA activities in WB and BB samples were significantly (*p* < 0.05) affected by US, GE, and US + GE, whereas non-significant (*p* > 0.05) effect was shown S. Similarly, H_2_O_2_ and O_2_^–^ generation markedly increased in US + GE treatment compared to others, Fig. 4 (E) and (F). Our findings are consistent with previous trends regarding the effect of stress implication on stress indicators [11,26,32,50,51].

### 3.8 Impact of different treatments on permeability and surface morphology of barley fractions

The relative conductivity (RC) index reflects cell membrane permeability. Usually, healthy plant cells have a lower RC index; however, physical stress can induce membrane damage in grains, increasing electrolyte leakage, enhancing RC Fig. 4 (G). The results revealed that the relative conductivity of WB and BB samples increased after exposure to physical stress, as cell membrane permeability increased significantly (p < 0.05) in US and US + GE treated fractions. However, it remained unchanged in GE and S-treated samples. Previously, similar results have been reported by [52].

**Fig. 5:**
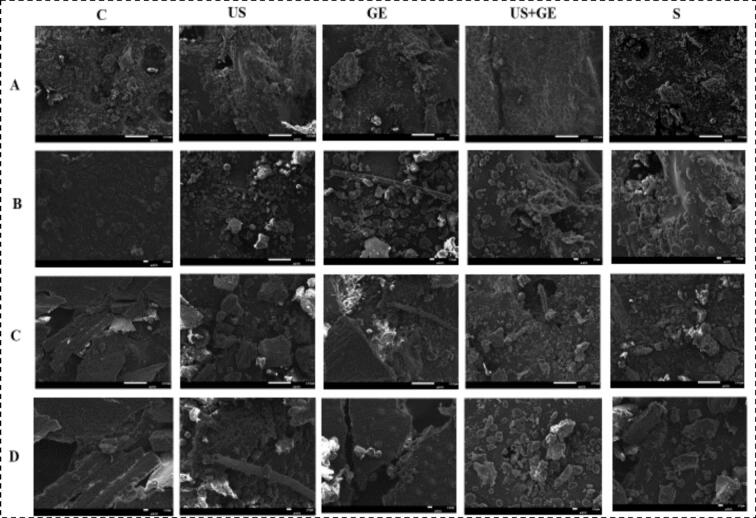
Morphological characterization of highland’s barley fractions subjected to ultrasonication, germination, ultrasound assisted germination and soaking. A. whole barley flour 200X. B. Whole barley flour 400X. C. Whole barley bran 200X D. Whole barley bran 400X. C: untreated barley, US: multifrequency ultrasonication, GE: germination, S: soaking.

Scanning electron microscopy (SEM) is commonly applied to characterize microstructural changes in cereal grains and cereal-based foods [53]. The SEM micrographs of treated barley fractions are illustrated in Fig. 5. As shown in Fig. 5, control WB showed a rough, irregular surface with a fractured structure, while the surface of BB was a compact, fibrous, and layered structure with an intact, rough surface. After the US, the SEM micrograms of WB showed a highly disrupted, porous, and fragmented surface structure, indicating the US has a disintegrated cell wall and increased surface exposure. In contrast, BB showed a disrupted, permeable, fractured surface structure with loosened fibrous layers.

GE-treated WB also showed a porous, fragmented structure with ruptured and swollen regions, whereas germinated BB showed a porous, loosened structure with fibre degradation. Furthermore, US + GE WB showed a highly fragmented, porous structure with extensive cell wall breakdown, enhancing the surface area and nutrient accessibility. In contrast, BB showed high porosity, fiber fragmentation, and cell wall disruption, suggesting that the combination of germination and ultrasonication has improved structural breakdown compared to separate treatments.

### 3.9 Impact of different treatments on the textural profile of barley fractions

**Fig. 6:**
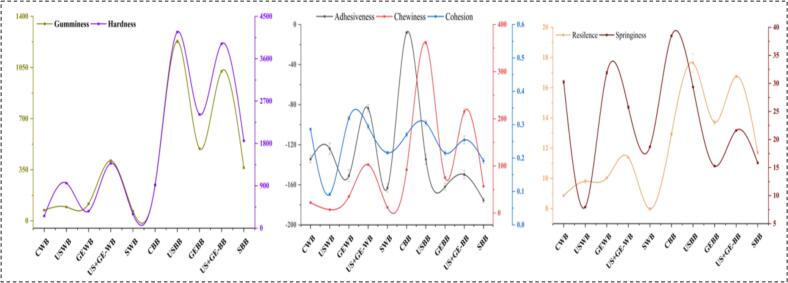
Texture profile analysis of highland’s barley fractions subjected to ultrasonication, germination, ultrasound assisted germination and soaking. A. Gumminess and hardness of treated barley fractions. B. adhesiveness, chewiness and cohesion of treated barley fractions. C. resilience and springiness of treated barley fractions. CWB: untreated barley flour, USWB: multifrequency ultrasonicated barley flour, GEWB: germinated barley flour, SWB: soaked barley flour, CBB: untreated barley bran, UBB: multifrequency ultrasonicated barley bran, GEF: germinated barley bran, SF: soaked barley bran.

Ultrasonication improves the texture of foods by modifying their protein structures, enhancing gelation, and influencing rheological properties like viscosity and elasticity. The process promotes uniformity in emulsions, reduces particle size, and enhances hydration, improving mouthfeel and consumer acceptability. The current research explored the effect of US, GE, and US + G on whole barley flour and barley bran depicted in Fig. 6. The results showed that the US significantly increases hardness, gumminess, and chewiness in both USWB and USBB, with the highest values in US BB.

Similarly, GEWB and GEBB showed increased hardness, gumminess, and cohesion, particularly in BB. However, S-treated WB and BB showed better results, indicating the softest texture, as illustrated in Fig. 6 (A). Moreover, US + GE- WB and US + GE- BB presented moderate texture modifications, balanced hardness, gumminess, adhesion, and cohesion. Adhesiveness was maximum in untreated CWB and CBB, whereas reduced chewiness and adhesion were observed in SWB and SBB, as shown in Fig. 6 (B). However, the US improved springiness in WB and BB, while GE enhanced resilience due to decreased elasticity, as shown in Fig. 6 (C).

Previously, Guo et al. [54] reported the effect of US-treated fresh and aged rice flour gel texture, showing similar results. Furthermore, US at 100 W for 120 min significantly improved the texture of aged rice gel by reducing hardness, chewiness, and gumminess, linked to US-induced starch granule disaggregation, enhanced water binding capacity, and increased short-range ordered and double helix structures in starch. Similarly, Yildirim et al. [55] reported an improved texture profile of ultrasonicated chickpea. Ultrasound treatments accelerated texture changes, reducing the time to reach equilibrium texture from 941 to 82 min as temperature increased from 20 to 97 °C. These findings suggest that ultrasonic treatments can soften chickpeas and shorten cooking times, benefiting industrial processing.

### 3.10 Impact of different treatments on the FTIR profile of barley fractions

**Fig. 7:**
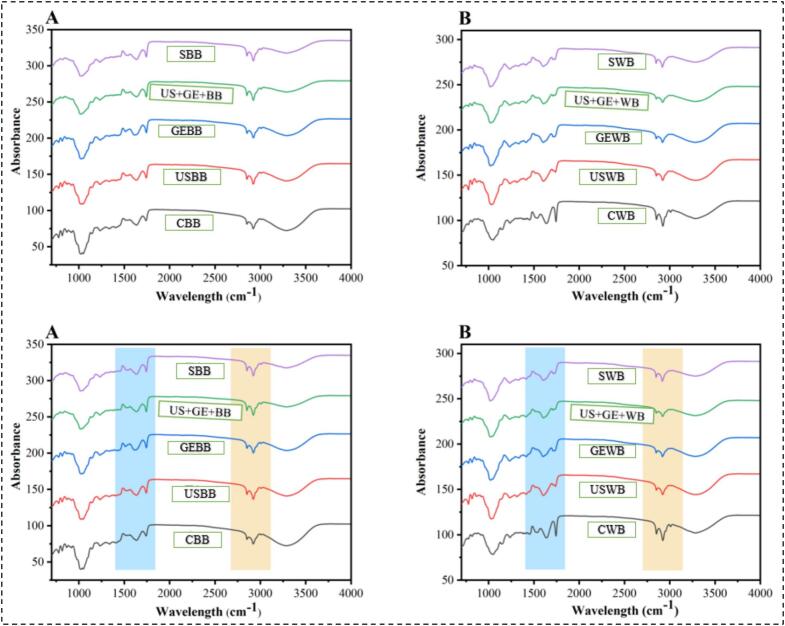
FTIR spectrums of highland’s barley fractions subjected to ultrasonication, germination, ultrasound assisted germination and soaking. CWB: untreated barley flour, USWB: multifrequency ultrasonicated barley flour, GEWB: germinated barley flour, SWB: soaked barley flour, CBB: untreated barley bran, UBB: multifrequency ultrasonicated barley bran, GEF: germinated barley bran, SF: soaked barley bran.

The structural transformations induced by US, GE, US + GE, and S in WB and BB were determined through FTIR spectra presented in Fig. 7. Each spectrum signifies distinct treatments; in Fig. 7, graph A shows the untreated barley (CWB), whereas USWB and USBB portrayed the most considerable structural alterations. The amide I band (1600–1700 cm^–^1), which is mainly linked to C=O stretching in proteins, suggests important facts about secondary structural changes introduced by analytical techniques. From Fig. 7 (A), controlled WB showed a remarkable amide I band (1600–1700 cm^–^1), which is mainly linked to proteins' C=O stretching vibrations. The peaks in this region indicate an unmodified protein structure, which demonstrates the presence of α-helix, β-sheet, and random coil structures. Various other leading peaks might be correlated with polyphenol chemicals in barley, such as carboxylate (COO^–^) stretching (1600–1650 cm^−1^) and aromatic C=C stretching (∼1610 cm^−1^). As an untreated sample, the peak intensities and positions are used as indicators to compare different treatments. Explicit peaks typical of native protein structures, such as α-helices (∼1650 cm^−1^), β-sheets (∼1630 cm^−1^), and random coil structures (∼1640–1660 cm^−1^) were visible in untreated WB and BB (C1, CB1). The study suggests that ultrasound treatment of barley gluten proteins boosts solubility and emulsification properties, similar to [47] outcomes. The use of US-enhanced protein rearrangements is more competent. Compared to the control (CWB), the FTIR spectra of USWB and USBB (graphs B & G) showed notable spectrum changes. Higher Peak Intensities are an apparent rise in absorbance of the amide I area (1600–1700 cm^–1^), which may reveal alterations in protein structure. Ultrasonic cavitation breaks hydrogen bonds and increases molecular mobility. Shift in Peak Positions protein unfolding or partial denaturation is revealed by slight shifts in the 1640–1660 cm^−1^ peaks associated with α-helix and random coil structures. This may boost bioavailability and digestibility. The US produced the most evident structural alterations in WB and BB compared to all other treatments, indicating higher molecular mobility and changed hydrogen bonding patterns. Combining germination with different processing methods boosts protein functionality, while the US independently offers a more efficient alternative to multi-step processing.

Compared to ultrasound-treated samples, the FTIR spectra of GEWB and GEBB (graphs C & H) unveil less obvious alterations. Intensity alternations in the amide I area reveal that germination causes partial hydrolysis of proteins. These modifications may be attributed to enzymatic alterations, including the breakdown of storage proteins into peptides. FUS-treated WB and BB enhanced the molecular rearrangement of barley proteins, directing them to more structurally progressive structures. When US + GEWB and US + GEBB (graphs D & I) are combined, the resulting spectrum characteristics include germination- and ultrasound-induced changes. Intermediate spectral characteristics were displayed by the combined treatment US + GEWB and US + GEBB, indicating synergistic but not superior benefits over the US alone. US-treated WB and BB (Graph B &G) showed the most noticeable spectrum alterations, suggesting that US alone is more suitable than combined treatment.

### 3.11 Impact of different treatments on the rheological properties of barley fractions

US significantly affects the rheological properties of cereal foods, including barley and barley bran, by modifying starch and protein structures. It enhances dough elasticity, viscosity, and water absorption, improving texture and fermentation characteristics. The cavitation effect alters gluten proteins, increasing solubility and foaming ability, leading to better-quality flour products. In barley-based products, the US improves gelling properties and hydration, enhancing the texture and functional quality of the final food product.

**Fig. 8:**
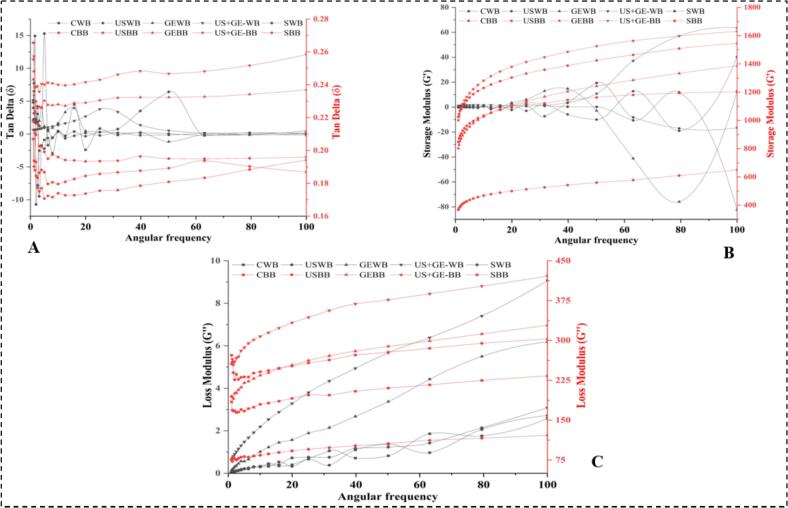
Rheology of highland’s barley fractions subjected to ultrasonication, germination, ultrasound assisted germination and soaking. A. Tan delta of treated barley fractions. B. Storage modulus of treated barley fractions. C. Loss modulus of treated barley fractions. CWB: untreated barley flour, USWB: multifrequency ultrasonicated barley flour, GEWB: germinated barley flour, SWB: soaked barley flour, CBB: untreated barley bran, UBB: multifrequency ultrasonicated barley bran, GEF: germinated barley bran, SF: soaked barley bran.

The rheological analysis showed that US + GEWB and US + GEBB significantly influence the tan delta (δ) values, indicating altered viscoelastic properties, as shown in Fig. 8 (A). The tan delta values for BB are generally lower than those for WB, suggesting that the US affected bran fraction differed, likely due to its higher fibre content.

GEWB and GEBB and US + GEWB and US + GEBB provided more stable than delta values across increasing angular frequencies, implying improved structural integrity. SWB and SBB resulted in the least variation in tan delta in Fig. 8 (A), indicating a less pronounced effect on viscoelastic properties than US and GE. Moreover, the US enhanced elasticity, with a pronounced impact on USBB, illustrated in Fig. 8 (B). GEWB, GEBB and US + GEWB and US + GEBB improved G's stability, while SWB and SBB had minimal impact.

However, USWB and USBB increased the loss modulus (G''), with USBB showing a more pronounced effect, indicating greater energy dissipation in BB, as shown in the Fig. 8(C). GE & and US + GE enhanced G'' stability in both fractions, reflecting improved structural interactions, while S showed a non-significant effect. Therefore, US and GE-treated fractions showed significantly altered viscoelastic and viscous properties.

Previously, similar results have been reported by [56], who explored the impact of ultrasound on wheat gluten. Results showed improved foaming capacity and stability with increasing treatment power, peaking at 100 %. Emulsifying capacity and stability were lowest at 60 % power, while electrophoretic patterns remained largely unchanged. Ultrasound influenced the storage loss moduli and showed a U-shaped alteration.

### 3.12 Impact of different treatments on crystallinity of barley fractions

**Fig. 9:**
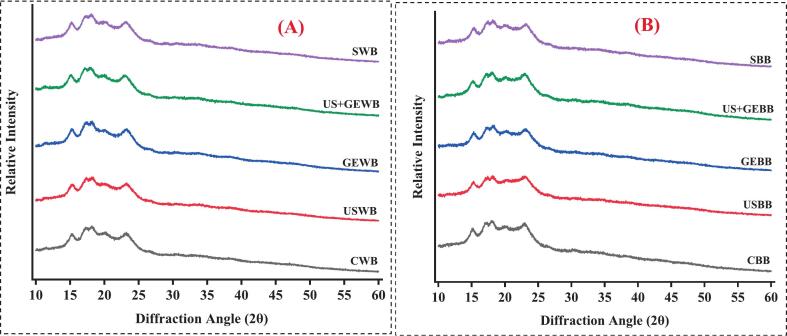
XRD analysis of highland’s barley fractions subjected to ultrasonication, germination, ultrasound assisted germination and soaking. (A) XRD of treated whole barley flour. (B) XRD of treated barley bran. CWB: untreated barley flour, USWB: multifrequency ultrasonicated barley flour, GEWB: germinated barley flour, SWB: soaked barley flour, CBB: untreated barley bran, UBB: multifrequency ultrasonicated barley bran, GEF: germinated barley bran, SF: soaked barley bran.

X-ray diffractions illustrate crystalline and amorphous structures of macromolecules; starch exhibits a semi-crystalline structure comprising a double helix of amylopectin, showing a distinct pattern in XRD, indicated by sharp peaks [57]. However, stress implication modifies starch crystallinity by affecting the molecular configuration of these molecules. The effect of US, GE, US + GE, and S on crystalline and amorphous structures of WB and BB is presented in Fig. 9. Two distinct diffraction peaks were observed in native barley flour. 1st peak was observed at 15.31°, whereas 2nd peak was observed at 23.32°. As evident from Fig. 9 (A), no significant molecular modification was observed in the case of S, as the peak pattern was similar to the control WB. However, the peak widens in other treated samples, and a maximum increase in peak width was observed in USWB, indicating high molecular modifications resulting from starch degradation contributing to the creation of amorphous regions. In combined treatment, US + GE peak shift was observed; 1st peak shifted from 15.31°-14.95°, and the 2nd peak shifted from 23.50°–22.80°. The increased peak width and moving to a lower angle in US + GEWB indicated that the combined treatment of US + GE increased amorphous regions by disrupting starch crystals.

Like CWB, CBB showed 1st peak at 15.06° and 2nd peak at 23°. In the case of BB, there was no peak shift; however, in all treated samples, peak intensity varied, as shown in Fig. 9 (B). USBB showed marked intensity reduction in the 2nd peak with increased width, indicating enhanced amorphous regions. A similar peak pattern was observed for US + GE, accompanied by a decrease in the intensity of the first diffraction peak, suggesting increased structural asymmetry and irregular molecular configuration. Contrary to WB, in SBB, both diffraction peaks widen. This could be linked to elevated water absorption due to higher fibre content, causing swelling and subsequent disruption of starch molecules, increasing amorphous regions*.* Combined treatment increases relative crystallinity of the starch compared to individual treatments, as ultrasonication facilitates increased water uptake and endogenous enzyme activity promoting germination efficiency [58,59]. Ultrasonication-assisted germination increases crystallinity of highland barley starch [60], whereas ultrasonication alone disrupts the amorphous region, ultimately increasing crystallinity of finger millet starch [61].

### 3.13 Impact of different treatments on the aromatic profile of treated barley fractions

**Fig. 10:**
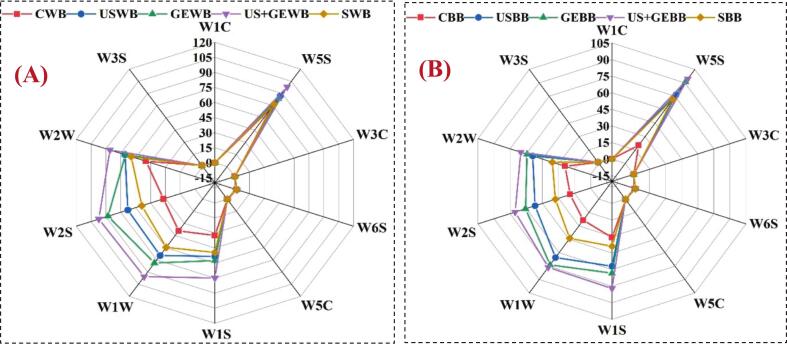
Aromatic profile of highland’s barley fractions subjected to ultrasonication, germination, ultrasound assisted germination and soaking. (A) E-nose profile of treated whole barley flour. (B) E-nose profile of treated barley bran. CWB: untreated barley flour, USWB: multifrequency ultrasonicated barley flour, GEWB: germinated barley flour, SWB: soaked barley flour, CBB: untreated barley bran, UBB: multifrequency ultrasonicated barley bran, GEF: germinated barley bran, SF: soaked barley bran.

The E-nose analysis revealed distinct differences in volatile compound profiles of barley fractions subjected to various treatments. The control WB and BB produced baseline sensor responses that were significantly different from those of the treated samples. USWB, USBB, US + GEWB and US + GEaBB showed highly significant changes in sensor responses, as indicated by their separation in PCA and cluster analyses, presented in Fig. 10 (A-C). These treatments appeared to induce significant alterations in the chemical composition of the barley fractions, particularly in terms of VOCs. Similarly, previous studies have shown that the US alters the aromatic profiles of plant materials by enhancing the release of VOCs [25].

On the other hand, GEWB, GEBB, SWB and SBB showed moderate changes, with sensor responses closer to control samples. These results align with prior work, suggesting that GE can enhance specific biochemical properties of grains; however, its effect on the aromatic profile may be less pronounced than in the US. Similarly, SWB and SBB showed minimal impact on the volatile profiles, which aligns with studies indicating that soaking alone does not significantly alter the chemical composition of grains.

Statistical analysis confirmed that US + GE produced significant differences in volatile profiles compared to the control, suggesting a substantial impact of processing on barley fractions, supporting the stance that the US and its combined treatments could enhance or modify the aroma and flavor profiles of barley fractions, potentially improving overall consumer appeal of barley-based products. These results are consistent with Sun et al. [26], demonstrating the potential of the US and its combined treatments in enhancing sensory attributes of food products.

## 4 Conclusion

Non-thermal processing pretreatment offers a promising approach to developing healthy and nutritious foods. Among these techniques, ultrasonication has demonstrated significant potential in enhancing the nutritional and functional properties of flour, barley, and barley bran. The present study revealed that ultrasound-assisted germination treatment significantly increased barley fractions' GABA accumulation, amino acid release, and antioxidant activity; furthermore, notable structural modifications as evidenced by SEM micrographs and FTIR spectra. Similarly, the textural and aromatic profiles of treated barley fractions were improved. These enhancements contribute to the suitability of barley and barley bran for various food processing applications. Therefore, either alone or in synergy, ultrasonication emerges as a promising non-thermal technology for improving the quality profile of barley fractions. Our studies highlight its potential for functional food implications; however, further optimization and pilot-scale studies are required to assess its industrial feasibility and economic viability.

**Funding Statement and Acknowledgement**

The authors wish to express their appreciation for the support obtained from the Suzhou Key Core Technology Research (Agriculture, Social Development) Project (SNG2023020) and Sihong County Science and Technology Innovation Project (H202317).

## CRediT authorship contribution statement

**Tabussam Tufail:** Writing – review & editing, Writing – original draft, Methodology, Investigation, Data curation, Conceptualization. **Huma Bader Ul Ain:** Writing – review & editing, Writing – original draft, Methodology, Investigation, Data curation. **Jawad Ashraf:** Writing – review & editing, Formal analysis, Data curation. **Farhan Saeed:** Writing – review & editing, Writing – original draft, Visualization, Supervision. **Zunaira Basharat:** Writing – review & editing, Methodology. **Zahoor Ahmed:** Writing – review & editing, Validation, Data curation. **Muhammad Waseem:** Writing – review & editing, Data curation. **Bin Xu:** Writing – review & editing, Visualization, Supervision, Resources, Project administration. **Muhammad Faisal Manzoor:** Writing – review & editing, Validation. **Robert Mugabi:** Writing – review & editing, Writing – original draft, Software.

## Declaration of competing interest

The authors declare that they have no known competing financial interests or personal relationships that could have appeared to influence the work reported in this paper.
